# How important are fatty acids in human health and can they be used in treating diseases?

**DOI:** 10.1080/19490976.2024.2420765

**Published:** 2024-10-27

**Authors:** Leon M. T. Dicks

**Affiliations:** Department of Microbiology, Stellenbosch University, Stellenbosch, South Africa

**Keywords:** Fatty acids, human health, treatment of diseases

## Abstract

Most of the short-chain fatty acids (SCFAs) are produced by *Bifidobacterium*, *Lactobacillus*, Lachnospiraceae, *Blautia*, *Coprococcus*, *Roseburia*, *Facealibacterium* and *Oscillospira*. Butyrate (C_4_H_7_O_2_^−^) supplies 70% of energy to intestinal epithelial cells (IECs), supports tight-junction protein formation, induces the production of inflammatory cytokines, and inhibits histone deacetylase (HDAC). Butyrate is also associated with the recovery of brain trauma, improvement of dementia, the alleviation of autoimmune encephalitis, and several intestinal disorders. Low levels of SCFAs are associated with hypertension, cardiovascular disease (CVD), strokes, obesity, and diabetes mellitus. *Cis-*palmitoleic acid (C_16_H_30_O_2_), a mono-unsaturated fatty acid (MUFA), increases insulin sensitivity and reduces the risk of developing CVD. Lipokine palmitoleic acid reduces the expression of pro-inflammatory cytokines IL-1β (pro-IL1β), tumor necrosis factor α (TNF-α), and isoleucine 6 (IL-6). Polyunsaturated fatty acids (PUFAs), such as omega-3 and omega-6, are supplied through the diet. The conversion of PUFAs by cyclooxygenases (COX) and lipoxygenases (LOX) leads to the production of anti-inflammatory prostaglandins and leukotrienes. Oxidation of linoleic acid (LA, C_18_H_32_O_2_), an omega-6 essential fatty acid, leads to the formation of 13-hydroperoxy octadecadienoic acid (13-HPODE, C_18_H_32_O_4_), which induces pro-inflammatory cytokines. Omega-3 PUFAs, such as eicosapentaenoic acid (EPA, C_20_H_30_O_2_) and docosahexaenoic acid (DHA, C_22_H_32_O_2_), lower triglyceride levels, lower the risk of developing some sort of cancers, Alzheimer’s disease and dementia. In this review, the importance of SCFAs, MUFAs, PUFAs, and saturated fatty acids (SFAs) on human health is discussed. The use of fatty acids in the treatment of diseases is investigated.

## Introduction

**Figure 1.**
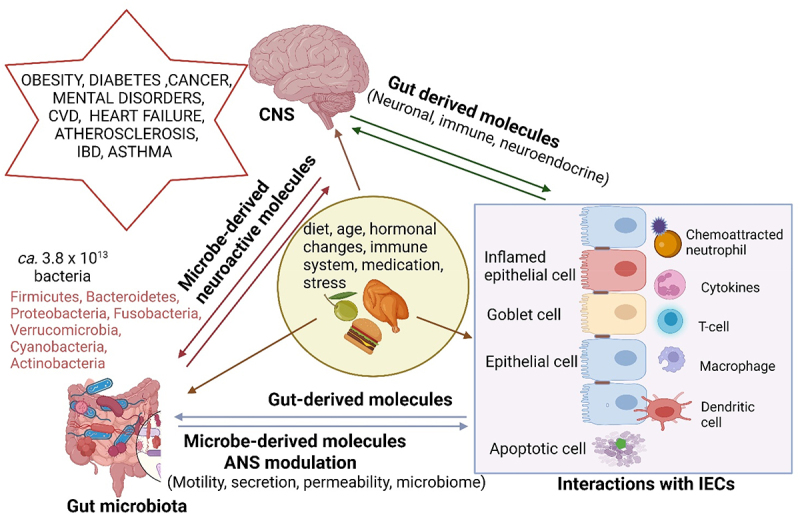
Gut microbiota, intestinal epithelial cells (IECs), the autonomic nervous system (ANS) and the brain (central nervous system, CNS) are in constant contact via bidirectional communication channels driven by gut- and microbe-derived molecules that have a direct or indirect effect on the formation of neuronal, immune, and neuroendocrine signals. These interactions regulate the composition of the gut microbiome, bowel movement, and the migration of molecules across the gut wall. Some microbe-derived molecules reach the brain via the vagus nerve or enter the systemic circulation system (bloodstream). Neuroactive molecules released from the brain affect the behavior of gut microbiota and their gene expressions. An imbalanced diet, obesity, diabetes, cancer, mental disorders, and microbial infections are examples of abnormalities that alter the composition of the gut microbiome. Metabolites produced by gut microbiota have also been implicated in some disease processes, such as cardiovascular disease (CVD). Created using Biorender.com (1 July 2024).

**Figure 2.**
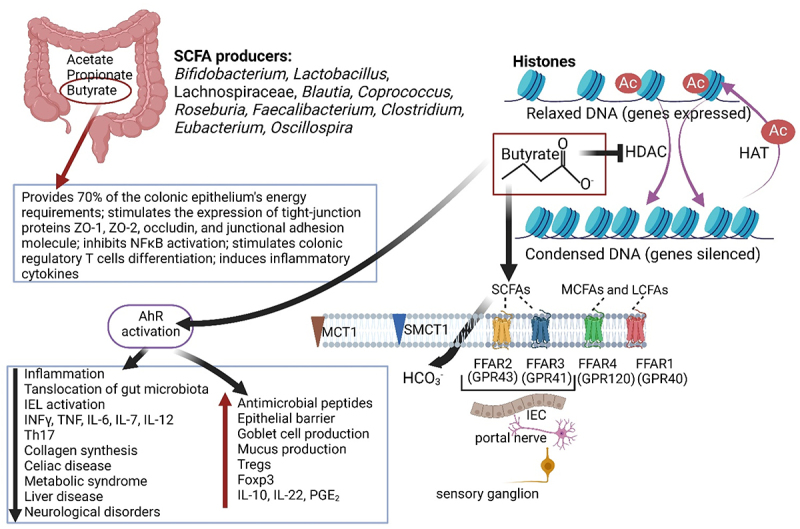
The role of short-chain fatty acids (SCFAs), especially butyrate, in inflammation, gene expressions, gut wall integrity, and disease. HDAC: histone deacetylase, HAT: histone acetyl transferase, MCFAs: medium-chain fatty acids, LCFAs: long-chain fatty acids, MCT1: monocarboxylate transporter-1, SMCT1: sodium-coupled monocarboxylate transporter-1, FFAR: free fatty acid receptor, GPR: G-protein receptor, IL: isoleucine, PGE2: prostaglandin E2, INFγ: interferon gamma, TNF: tumor necrosis factor, Th17: T-helper cell 17, nFƙB: nuclear factor kappa-B. Created using Biorender.com (1 July 2024).

The adult human gut is host to approximately 3.8 × 10^13^ (0.2 kg) bacteria, more or less equivalent to the estimated 3.0 × 10^13^ cells in a person of 70 kg.^1^ Most gut bacteria belong to the phyla Bacillota (Firmicutes) and Bacteroidota (Bacteroidetes)^2^ but are also represented by Pseudomonadota (Proteobacteria), Fusobacteriota (Fusobacteria), Verrucomicrobiota (Verrucomicrobia), Cyanobacteria, and Actinomycetota (Actinobacteria) (Figure 1). To a large extent, gut microbiota regulates the uptake of macronutrients^3,4^ but their development is controlled by diet, age, hormonal changes, the host’s immune system,^5^ and external factors such as medication, and stress (Figure 1). Western-style diets high in animal proteins have been associated with cardiovascular diseases (CVDs) such as atherosclerosis and heart failure but also obesity, type 2 diabetes mellitus,^6–8^ irritable bowel disease, IBD, and asthma (Figure 1). A low protein or Mediterranean diet (MD) with plant-based products such as fruit, nuts, oils, and seeds^9^ contains more unsaturated fatty acids and is considered healthier with fewer reports of CVDs, insulin resistance, and an imbalance in immune responses.^10,11^ The gut microbiome of humans on an MD is dominated by *Bifidobacterium*, *Enterococcus*, *Prevotella*, *Bacteroides*, *Faecalibacterium prausnitzii*, *Roseburia*, and *Lachnospiraceae*.^12,13^ However, low cell numbers of *Ruthenibacterium lactatiformans*, *Flavonifractor plautii*, *Parabacteroides merdae*, *Ruminococcus torques*, and *Ruminococcus gnavus* were reported.^14^ An increase in *Lactobacillus* ^12^ and Firmicutes was also noted^15^ (Figure 2).

Diets high in fiber support the growth of glycan-degrading gut microbiota and the production of short-chain fatty acids (SCFAs) such as butyrate (C_4_H_7_O_2_^−^), propionate (C_3_H_5_O_2_^−^), and acetate (C_2_H_3_O_2_^−^).^16^ Fructan and galactooligosaccharide (GOS)-rich diets stimulate the growth of *Bifidobacterium* and *Lactobacillus*.^17^ Some researchers claim that the consumption of grains stimulates the production of phenolic compounds that promote the growth of bifidobacteria.^18^ These findings were, however, not confirmed when oats were the staple diet, as shown by Kristek *et al*.^19^ Neither beta-glucans nor polyphenols stimulated the growth of bifidobacteria. It is important to support the growth of bifidobacteria and lactic acid bacteria, as they produce several SCFAs that have probiotic properties.^20,21^ According to McDonald *et al*,^22^ the gut microbiome of individuals who consumed more than 30 plant types weekly is dominated by SCFA producers, including *F. prausnitzii* and *Oscillospira* spp. The growth of these species is stimulated by acetate-producing *Bifidobacterium* and *Akkermansia*.^23^

High-molecular-weight beta-glucans stimulated the growth of Bacteriodetes and *Prevotella*, and repressed the growth of Firmicutes and *Dorea*.^24^ This was not observed with a diet of low-molecular-weight beta-glucans.^24^ In rats, beta-glucans from oats led to an increase in *Lactobacillus* and *Bifidobacterium* but a decrease in Enterobacteriaceae.^25^ In pigs, an oat diet led to an increase in *Lactobacillus, Streptococcus, Enterococcus*, *Clostridium* clusters I and XIVa, certain species of *Bacteroides, Prevotella*, *Porphyromonas*, and Enterobacteriaceae.^26^ Arabinoxylans have been associated with an increase in *Bifidobacterium animalis* subsp. *lactis*, *Prevotella, F. prausnitzii*, and *Lactobacillus*, but a decrease in *Escherichia. coli, Streptococcus, Staphylococcus, Lactobacillus, Clostridium histolyticum* I and II, and *Enterococcus* .^18^ Long-chain arabinoxylans also promoted the growth of *Bifidobacterium longum* with a concurrent increase in propionate levels.^27^

In this review, the importance of SCFAs, monounsaturated fatty acids (MUFAs), polyunsaturated fatty acids (PUFAs), and saturated fatty acids (SFAs) on human health is discussed. The option of using fatty acids in the treatment of diseases is also investigated.

## Short-chain fatty acids (SCFAs)

Most SCFAs are produced in the colon by *Bifidobacterium, Lactobacillus*, Lachnospiraceae, *Blautia, Coprococcus, Roseburia, Faecalibacterium, Clostridium*, and *Eubacterium*.^28,29^ Of all SCFAs, butyrate is the best studied, as it supplies 70% of the energy requirements of the colonic epithelium,^30^ plays a critical role in the expression of tight-junction proteins ZO-1, ZO-2, occludin, and junctional adhesion molecule A,^31^ and has direct anti-inflammatory effects, inhibiting nuclear factor kappa-B (NFκB) activation (Figure 2). Butyrate also stimulates the differentiation of colonic regulatory T cells,^32^ and induces inflammatory cytokines (Figure 2).

SCFAs affect at least two systems of molecular signaling that have widespread regulatory effects, i.e., the deacetylation of histones, regulated by histone deacetylase (HDAC), and the adherence to G-protein-coupled receptors (GPCRs), also called free fatty acid receptors (FFARs) (Figure 2). G-protein receptor 43 (GPR43/FFAR2) and GPR41 (FFAR3) are located on the surface of intestinal epithelial cells (IECs),^32^ neurons of the enteric nervous system (ENS), portal nerve, and sensory ganglia,^33,34^ as shown in Figure 2. GPR43, mostly expressed in subcutaneous fat, visceral fat, and bone marrow, regulates energy expenditure in skeletal muscles and in the liver.^35^ GPR 41, activated by propionic acid (C_3_H_6_O_2_),^36^ transfers signals directly to the central nervous system (CNS)^37^ and induces the nuclear phosphoprotein Fos in the dorsal vagal complex of the brainstem, the hypothalamus, and the spinal cord.^38^ FFAR4 (GPR120) is expressed in adipocytes, endothelial cells, and macrophage^39^ and assists in the regulation of adipogenesis, insulin sensitivity, and inflammation. Dysfunction of FFAR4 is associated with insulin resistance, obesity, and eccentric remodeling.^39^ FFAR1 (GPR40) senses long-chain free fatty acids (FFAs) produced by lipolysis and endogenously synthesized triglycerides.^40^ The binding of FFAs to FFAR1 on pancreaticβ-cells and enteroendocrine cells activates signaling through the transducer protein Gq and β-arrestin.^40^ This releases Ca^2+^ into the cytosol that activates protein kinase C, which enhances the release of insulin and glucose uptake.^40^ Apart from regulating energy levels, FFAR1 also plays a role in regulating pain and inflammation in the brain.^40^ Most SCFAs are transported across the gut wall in dissociated form by an HCO_3_^−^ exchanger of unknown identity, a monocarboxylate transporter-1 (MCT1) or sodium-coupled monocarboxylate transporter-1 (SMCT1) (Figure 2). Some SCFAs, however, diffuse across IEC membranes and enter the bloodstream in a non-ionized form.^41^ It is also noteworthy that SCFAs stimulate antimicrobial peptides through the cathelicidin LL-37 pathway, as shown in the prevention of *Shigella* infections.^42^

The acetylation and deacetylation of histones is a fundamental process in DNA coiling and the regulation of gene expression. Butyrate acts as an HDAC inhibitor (HADCi), thus preventing the deacylation of histones (Figure 2) and increasing the expression of repressed genes.^43^ This process is crucial in activating extrinsic and intrinsic apoptotic pathways, reactive oxygen species (ROS), and cell cycle arrest in cancer cells.^44–46^ The inhibition of HDAC also impacts several other diseases, such as brain trauma, dementia, and autoimmune encephalitis.^47,48^ By inhibiting HDAC, chromatin is exposed to aryl hydrocarbon receptor (AhR)-ligand complexes and binding sites in the promoter of AhR target genes. Butyrate thus modulates AhR activation.^49^ Binding to Ahr is important in several metabolic and immune processes (Figure 2), allowing the co-existence of gut microbiota and their host.^50^ The activation (increase) of AhR downregulates intestinal inflammation, alleviating inflammatory bowel diseases (IBD), including Crohn’s disease and ulcerative colitis (UC), but also celiac disease, metabolic syndrome, liver disease, and neurological disease, as summarized in Figure 2. Elevated levels of AhR lead to a decrease in IFNγ, IL-6, IL-12, TNF, IL-7, and IL-17, a decline in microbial translocation and fibrosis, an increase in regulatory mechanisms such as IL-10, IL-22, prostaglandin E_2_, and Foxp3 (scurfin), the production of antimicrobial peptides, and the restitution of damaged epithelial cells, as listed in Figure 2. An increase in deacetylated histones decreases the expression of pattern recognition receptors, kinases, transcription regulators, cytokines, and chemokines. In mice, the inhibition of HDACi in the frontal cortex and hippocampus alleviated depressive behavior,^51^ dementia, and brain trauma.^52^ Patients suffering from neurological disorders such as depression, Parkinson’s disease (PD), and schizophrenia, have higher than normal levels of HDAC.^53^ Parkinson’s disease is associated with increased cell numbers of enterobacteria and potentially harmful pro-inflammatory *Proteobacteria*, especially *Ralstonia*, and a decrease in *Prevotella*^53,54^ and butyrate-producing *Blautia*, *Coprococcus*, and *Roseburia*.^55,56^ In severe cases of PD, changes in the integrity of the blood-brain barrier (BBB), CNS functioning, and microglia maturation were observed.^57,58^ Studies conducted on germ-free (GF) mice have shown that defective microglia could be stimulated by supplementing the feed with butyrate, propionate, and acetate.^59^ Acetate crosses the BBB and accumulates in the hypothalamus.^60,61^ This stimulates the production of gamma-aminobutyric acid (GABA) in the brain.^62^ GABA is the most abundant neurotransmitter in the CNS of mammals and is co-transmitted with acetylcholine (ACH).^63^ An increase in ACH increases the expression of *BDNF*, encoding brain-derived neurotrophic factor (BDNF) in the frontal cortex and hippocampus.^64^ This stimulates brain development.^65^ Low levels of BDNF are associated with depression and anxiety.^66,67^ Neurological disorders may, thus, be prevented by keeping SCFAs and HDAC at optimal levels.

SCFAs and tryptophan precursors interact with receptors on the gut wall, muscle layers surrounding the gut, liver, pancreas, adipose tissue, and immune cells.^68^ In entero-epithelial cells (EECs), SCFAs stimulate the release of gut hormones^69^ and modulate genes encoding the cyclic adenosine monophosphate (cAMP) response element-binding (CREB) protein. The latter regulates the synthesis of catecholamine neurotransmitters such as dopamine (DA).^70,71^ With an increase in the expression of tyrosine hydroxylase and a decrease in DA-β-hydroxylase (DBH; EC 1.14.17.1), DA is converted to norepinephrine (NE).^72,73^ Elevated levels of DA caused by a deficiency in DBH may have a detrimental effect on the autonomic nervous system (ANS) that controls blood pressure and body temperature. In immune cells, SCFAs regulate T-regulatory cell differentiation^59,74^ and the maturation of microglial cells.^75^ Butyrate also activates ornithine decarboxylase, which results in the inhibition of polyamine metabolism and the activation of alkaline phosphatase.^76^

Low levels of SCFA have been associated with high blood pressure (hypertension), CVDs, strokes, obesity, and diabetes mellitus.^77^ In rats, hypertension could be prevented by restoring acetate levels in the cecum.^33,76^ Propionate administered to patients with obesity enhanced gut hormone secretion while reducing adiposity and overall weight gain.^77,78^ Propionic acid also inhibits NFκB and may improve insulin sensitivity by activating peroxisome proliferator-activated receptor gamma.^79^ However, despite the anti-inflammatory effects of propionic acid,^79^ it may have neurotoxic side effects, as reported for autism.^80^

SCFAs, produced by microorganisms, play a key role in microbiota-gut-brain axis (GBA) communication, protection of the intestinal barrier, and inflammatory responses. Levels of SCFAs, however, need to be carefully controlled, as several disadvantages have been reported. Acetate, for instance, promotes the production of intestinal IgA,^81^ stimulates the secretion of cytokine IL-6, and increases neutrophil recruitment.^35^

## Monounsaturated fatty acids (MUFAs) and polyunsaturated fatty acids (PUFAs)

### Monounsaturated fatty acids

**Figure 3.**
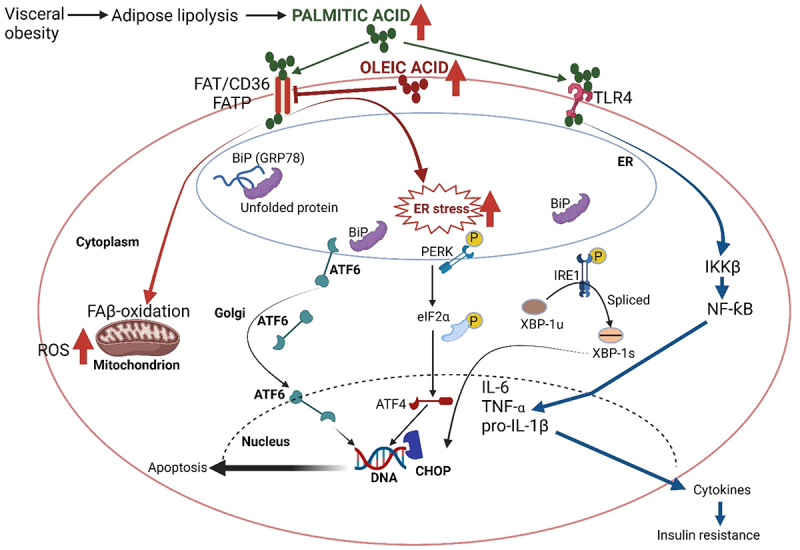
Visceral obesity and adipose lipolysis lead to the production of non-esterified fatty acids such as palmitic acid (C_16_H_32_O_2_). Stress induced on the mitochondrion and endoplasmic reticulum (ER) by palmitic acid results in fatty acid β (FAβ)-oxidation, an increase in reactive oxygen species (ROS), and apoptosis. Oleic acid (C_18_H_34_O_2_) represses the fatty acid translocase protein (FATP) FAT/CD36 and prevents an increase in ROS. Palmitic acid also triggers the transmembrane kinase protein (PERK) in the ER, which dimerizes and autophosphorylates, leading to the phosphorylation of the α subunit of eukaryotic initiation factor 2 (eIf2α) and induction of transcription factor 4 (ATF4) plus the CAAT/enhancer binding protein homologous transcription factor (CHOP), also known as GADD153, in the nucleus. CHOP is involved in DNA damage, growth arrest, and the induction of apoptosis. Under normal conditions, the three critical transmembrane proteins PERK, IRE-1 (inositol-requiring enzyme type 1), and ATF6 (an er-membrane-bound transcription factor) are associated with the major ER chaperone bip (GRP78) of the heat shock protein 70 family. Bip interacts with nonglycosylated and glycosylated proteins and er-transmembrane signaling molecules. Under ER stress conditions, bip is released and interacts with unfolded or misfolded proteins in the ER lumen. The autophosphorylation of IRE1 leads to the splicing of 26 nucleotides from the XBP1 (a transcription factor) mRNA. The smaller spliced XBP1 (XBP-1s) also promotes the transcription of CHOP. During ER stress, ATF6 is released from bip and translocates to the Golgi where it is proteolytically activated. The perk-eIf2α-ATF4-chop pathway plays an essential role in palmitic acid-triggered apoptosis. The suppression of ER stress by oleic acid and regulation of unfolded protein responses is important in preventing apoptotic cell death, especially in pancreatic β cells. Palmitic acid stimulates pro-inflammatory responses in human immune cells via Toll-like receptor 4 (TLR4). The degradation of IKKβ (IκB kinase β) activates nf-κB (nuclear factor kappa B), which induces the expression of various pro-inflammatory genes, including those encoding cytokines and chemokines. Created using Biorender.com (1 July 2024).

Monounsaturated fatty acids (MUFAs) are found in several plants, including olives, macadamia nuts, canola seeds, avocados, pumpkin seeds, sesame seeds, almonds, cashews, peanuts, and pecans. MUFAs contain a single double bond, whereas PUFAs contain two or more double bonds. Typical examples of MUFAs are palmitoleic acid (C_16_H_30_O_2_) or palmitoleate, also referred to as cis-9-hexadecenoic acid and oleic- or 9-octadecanoic acid (C_18_H_34_O_2_). Palmitoleic acid is formed in the liver when stearoyl-CoA desaturase (SCD-1) removes two hydrogen atoms from palmitic acid (C_16_H_32_O_2_) at the C-9 and C-10 positions.^82^ Palmitoleate is present in the *cis* (16:1c9) or a *trans* (16:1t9) isomer. The *cis* isoform (*cis*-palmitoleate) is associated with increased insulin sensitivity and less lipid accumulation in the liver.^83^ In animal models, *cis*-palmitoleate repressed the expression of proinflammatory markers and adipokines, and increased carbohydrate intake and lipogenesis.^84^ *Trans*-palmitoleate, found in dairy products and partially hydrogenated oils, is not strongly associated with incident diabetes^85^ nor linked to blood clotting or strokes.^86^ Palmitoleate, converted from palmitic acid, increases insulin sensitivity (Figure 3), and reduces the risk of atherosclerosis and CVD.^87,88^ Lipokine palmitoleic acid has anti-inflammatory properties and reduces the expression of pro-inflammatory cytokines IL-1β (pro-IL1β), TNF-α, and IL-6 (Figure 3). *In vitro* studies showed that palmitoleic acid reduced lipopolysaccharide (LPS)-induced inflammation in macrophages via inflammasome and NFκB pathways.^89^ High concentrations of palmitoleic acid (more than 50 mm) are toxic and lower concentrations reduce human peripheral blood lymphocyte proliferation, and T helper (Th1) and Th17 responses.^90^ Schirmer *et al*.,^91^ however, did not report a palmitoleic acid effect on lymphocyte-associated cytokines (IFNγ, IL-17, IL-22) when studied using human peripheral blood mononuclear cells (PBMNCs). The discrepancy between these findings may be due to the use of different cell populations, i.e., isolated lymphocytes versus PBMNCs.^90,91^ More research is required to understand the effect MUFA has on lymphocyte responses.

The effect of palmitic acid on reactive oxygen species (ROS) and apoptosis is schematically represented in Figure 3. Palmitic acid induces stress on mitochondria and the endoplasmic reticulum (ER), resulting in an increase in ROS and apoptosis.^92,93^ Oleic acid, in turn, prevents an increase in ROS. Under normal conditions, the three critical transmembrane proteins PERK (ER-resident transmembrane protein kinase), IRE-1 (inositol-requiring enzyme type 1), and ATF6 (ER-membrane-bound transcription factor) are linked to the major ER chaperone Bip (GRP78). Under ER stress conditions, Bip is released to interact with unfolded or misfolded proteins in the ER lumen.^94^ Triggering of PERK in the ER initiates the phosphorylation (activation) of the eukaryotic initiation factor 2α (eIF2α) and the induction of transcription factor ATF4 as well as the CAAT/enhancer binding protein homologous transcription factor (CHOP). The latter is involved in DNA damage, growth arrest, and the stimulation of apoptotic cell death. The autophosphorylation of IRE1 leads to the splicing of 26 nucleotides from the XBP1 mRNA. The XBP1 protein is a transcription factor that regulates gene expression in immunity and cellular stress response. The shorter spliced XBP1 (XBP-1s) also promotes the transcription of CHOP. When the ER is under stress, ATF6, released from Bip, is translocated to the Golgi and activated.^95^ ATF6 is an important signal transducer in cellular reprogramming that responds to protein misfolding in the endoplasmic reticulum. The mechanism by which ATF6 senses unfolded proteins and becomes activated is unknown.^96^ The alleviation of ER stress by oleic acid and regulation of unfolded protein responses are important in preventing apoptotic cell death, especially in pancreatic β cells.^97^ Palmitic acid also stimulates pro-inflammatory responses in human immune cells via Toll-like receptor 4 (TLR4).^98^ The degradation of IKKB (IκB kinase) activates NFκB.^99^ NFκB induces the expression of various pro-inflammatory genes, including those encoding cytokines and chemokines (Figure 3), and also participates in inflammasome regulation.^100^

### Polyunsaturated fatty acids and their synthesis

**Figure 4.**
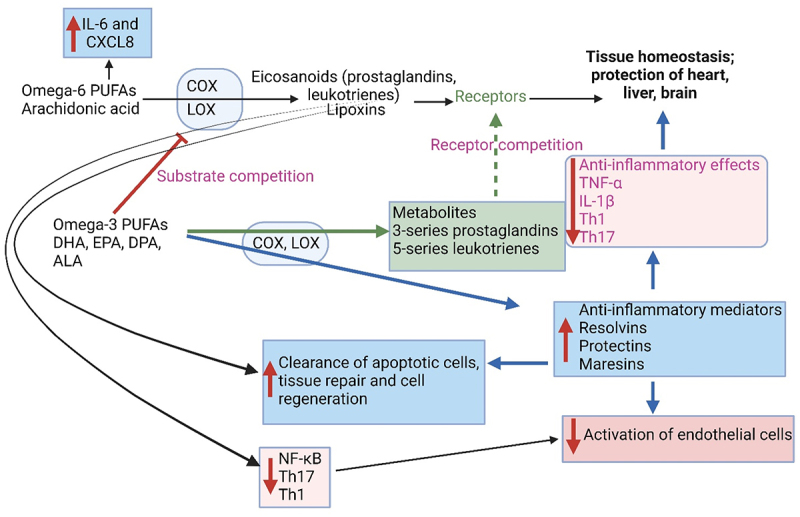
Omega-3 polyunsaturated fatty acids (PUFAs) and omega-6 PUFAs play a role in inflammation, the activation of endothelial cells, apoptosis, cell repair, and cell regeneration. IL: interleukin, CXCL8: C-X-C motif chemokine ligand 8, NFκB: nuclear factor kappa B, Th: T-helper cell, TNFα: tumor necrosis factor-alpha, CPT1A: carnitine palmitoyltransferase 1A, COX: cyclooxygenases, LOX: lipoxygenase, DHA: docosahexaenoic acid, EPA: eicosapentaenoic acid, DPA: docosapentaenoic acid, ALA: α-linolenic acid (C_18_H_30_O_2_). Created using Biorender.com (1 July 2024).

Polyunsaturated fatty acids (PUFAs), such as omega-3 and omega-6, are not produced in the body but form an essential part of a diet.^101^ Fish oil is rich in omega-3 fatty acids such as eicosapentaenoic acid (EPA) and docosapentaenoic acid (DPA), whereas α-linolenic acid (ALA; C_18_H_30_O_2_), an essential omega-3 fatty acid (Figure 4), is found in flaxseed oils.^102^ Several bioactive mediators derived from omega-3 PUFAs are involved in the recovery of injured and infected tissue cells (summarized in Figure 4). Cell debris and bacterial cells are phagocytized by polymorphonuclear leukocytes (PMNs), which are subsequently removed by recruited monocyte-derived macrophages. These reactions are orchestrated by anti-inflammatory prostaglandins and leukotrienes produced from the conversion of PUFAs by COX and LOX (Figure 4). Prostaglandins and leukotrienes are precursors of eicosanoids, i.e., signaling molecules regulating inflammation.^102^ Protectin, derived from DPA (Figure 4), represses the interactions between neutrophils and endothelial cells, neutrophil chemotaxis, and recruitment but increases macrophage phagocytosis.^102,103^ Protectins reduce the production of inflammatory cytokines, including MCP-1/chemokine C-X-C motif ligand-2 (CXCL-2).^104^ Maresin 1, also derived from DPA (Figure 4), stimulates macrophage phagocytosis and the clearance of human apoptotic neutrophils, similar to maresin-1 derived from EPA.^105^ Concluded from these and other findings,^106^ the biological effects displayed by EPA and DHA also apply to DPA. DPA incorporates inflammatory cells more easily than EPA and DHA and displays stronger anti-inflammatory properties.^107^ Omega-3 PUFAs may thus control inflammation by mediating molecules with low or no inflammatory activity.^108^ Omega-3 PUFAs have also been used in treating dyslipidemic disorders, thrombosis, atherosclerosis, and myocarditis.^108^ An increase in the consumption of omega-3 PUFAs altered the composition of gut microbiota, led to lower levels of LPS produced, and decreased intestinal permeability.^109^ DHA favors the proliferation of alpha gut bacteria, especially Lachnospiraceae^110^ and *Lactobacillus*.^111^ PUFAs significantly increase cell numbers of *Bifidobacterium*, *Lactobacillus*, and *Roseburia*.^111,112^

Pregnant rodents fed high levels of omega-3 led to a decrease in numbers of Lachnospiraceae, *Anaerotruncus*, and *Roseburia* and an increase in *Blautia, Oscillibacter*, Clostridiales, *Robinsoniella, Lactococcus*, and *Eubacterium* in offspring.^113^ The offspring of mice fed high levels of omega-3 fatty acids had lower levels of Bacteroidetes and higher levels of Firmicutes.^113^ In animal and human studies, a deficiency in omega-3 fatty acids early in life leads to diminished cognitive abilities, weakened attention, loss of vision, and psychological disorders such as depression, schizophrenia, and dementia.^109^ These conditions may be prevented when breastfeeding mothers take omega-3 fatty acid supplements.^109^ According to the authors, fatty acids in breast milk are only transferred to male infants. Omega-3 fatty acids are associated with improved metabolism and less weight gain in offspring.^109^ A reduction in maternal omega-3 acids is associated with a significant reduction in epsilon proteobacteria, *Bacteroides*, and *Akkermansia* but an increase in clostridia.^109^ Trans-10, cis-12 conjugated LA (t10-c12 CLA) in dairy products and red meat, and produced by *Lactobacillus plantarum* PL62, have antiobesity properties but may induce hepatic steatosis and hyperinsulinemia, specifically in diabetic or obese individuals.^114^ In mice, t10-c12 CLA reduced the Firmicutes:Bacteroidetes (F:B) ratio and decreased levels of Desulfovibrionaceae, Lachnospiraceae, Peptococcaceae, and Clostridiales Family XIII but increased Porphyromonadaceae.^115^ High-fat palm oil and high-fat olive oil diets led to obesity without a drastic change in gut microbiota composition. Diets rich in palm oil contain phytochemicals, lauric acid, retinoids, tocotrienols, and carotenoids. β-carotene in palm oil enhances gut immune homeostasis by modulating the production of IgA.^116^ Hidalgo *et al*.^117^ did, however, report an increase in Bacteroidetes when mice were fed olive oil but not when fed palm oil. This is interesting, as Bacteroidetes are associated with obesity. A high-fat palm oil diet, however, increased the F:B ratio, especially *Clostridium* clusters XI, XVII, and XVIII.^118^

Omega-6 arachidonic acid (AA, C_20_H_32_O_2_) is converted by COX and LOX to potential inflammatory mediators (eicosanoids; Figure 4).^102^ Omega-6 PUFAs are precursors of many pro-inflammatory signaling molecules that trigger inflammation.^108^ In the case of pulmonary infections, AA initiated the release of IL-6 and CXCL8. Cytokines produced by pulmonary fibroblasts are regulated by prostaglandin and p38 mitogen-activated protein (MAP) kinase signaling.^102^ Elevated levels of omega-6, typically found in a Western-style diet, may lead to more severe inflammation.^119^ Lipoxins (LX), also produced by the interaction of LOX with AA, (Figure 4) present anti- and pro-inflammatory reactions.^120^ *In vitro* tests have shown that LX reduces neutrophil migration^121^ and reduces inflammation in septic cells.^122^ *In vivo* studies have shown that LX increases neutrophil clearance.^121^ Lipoxin A4 (LXA4) regulates leukocyte tracking and responses,^123^ modulates the activation of vascular, smooth muscle, and epithelial cells,^124^ and reduces renal fibrosis.^125^ Binding of L×A4to the LX receptor (ALX) modulates the expression of adhesion molecules through inhibition of the NFκB pathway in endothelial cells.^126,127^

Omega-3 fatty acids have anti-inflammatory properties, whereas omega-6 fatty acids (not produced by humans) are pro-inflammatory.^128^ A balance between the two omega fatty acids is thus important to keep gut microbiota in a balanced state.^128^ The oxidation of linoleic acid (LA, C_18_H_32_O_2_), an omega-6 essential fatty acid, to 13-hydroperoxy octadecadienoic acid (13-HPODE), stimulates the production of TNF-α, MCP-1, IL-6 (pro-inflammatory cytokines) and cellular apoptosis.^128^ At the same time, barrier-forming tight junction proteins (TJPs) such as Claudin-1 and Occludin are downregulated, and pore-forming Claudin-2 is upregulated.^128^ This process, called “claudin switching”,^129^ leads to changes in the barrier function of the gut wall (IEC) and is often associated with IBD.^128,130^ The “switching” of TJPs is due to cytokine-mediated dysregulation.^129^ An increase in cytokine levels and a decrease in gut permeability were noted after 4 h when mice were fed 13-HPODE.^130^ After 28 days of 13-HPODE feeding, an increase in cholesterol uptake by peritoneal macrophages was noted, which was considered an indication of severe intestinal inflammation.^130^ PUFAs are metabolized by cyclooxygenase, lipoxygenase, and cytochrome P450 (CYP-450) to eicosanoids, lipoxygenases, and other essential metabolites (Figure 4). Linoleic 9,10-epoxy octadecenoic acid (9,10-EpOME or leukotoxin) and 12,13-epoxy octadecenoic acid (12,13-EpOME or iso-leukotoxin) are the main products derived from the metabolism of LA. Both variations of epoxy octadecenoic acids have immunomodulatory properties. Experiments with mice have shown a reduction in EpOMEs and dihydroxy octadecenoic acids (DiHOMEs) when fed a high-fat diet supplemented with the omega-3 α-linolenic acid (ALA).^131^ This also led to a lowering in the omega-6:omega-3 ratio, a decline in NFκB activation, divergence of M1 macrophages, and insulin resistance.^131^ 12,13-DiHOME increased Th2 cells, which increased the risk of developing asthma.^132^ In children suffering from asthma, the cell numbers of *Candida* and *Rhodotorula* spp. increased and those of *Bifidobacterium*, *Akkermansia*, and *Faecalibacterium* spp. decreased. The role of ALA in the differentiation of M2 macrophages is poorly understood. A recent study^133^ has shown that 13-hydroxy9(Z),15(Z)-octadecadienoic acid (13-OH), and 13-oxo-9(Z),15(Z)-octadecadienoic acid (13-oxo) produced by lactic acid bacteria regulates M2 differentiation. This is orchestrated via the GPCR40-MAPK and PPARγ signaling pathways in the presence of IL-4 and IL-13. Mice fed ALA, 13-OH, or 13-oxo for three days showed differentiation of M2 macrophages but only in the lamina propria of the small intestinal tract. No additional formation of adipose tissue, gut-associated lymphoid tissue, and mesenteric lymph nodes was observed.^133^

Studies conducted by Valenzuela *et al*. (2023)^134^ on Balb/c mice have shown that the highest levels of PUFA, based on the levels and activity of desaturases Δ-6D and Δ-5D, and elongases Elovl2 and Elovl5, were synthesized in the liver. Omega-3 and omega-6 PUFAs are desaturated by Δ-6 desaturase (Δ-6D) and Δ-5D, respectively, whereas the elongation of omega-3 and omega-6 PUFAs is regulated by elongases 2 (Elovl2) and Elovl5, respectively.^135^ In mice, low levels of PUFA were synthesized in the brain, testicles, and kidney and no PUFA enzyme activity was reported in the heart and lung.^134^ The production of Δ-5D in the liver was 4.3- to 22.9-fold higher (based on protein concentration and enzyme activity) compared to Δ-5D levels in the testicle.^134^ This compared to Elovl2 levels in the kidney.^134^ Furthermore, 4.0- to 85-fold higher levels of Δ-5D activity were observed in the liver compared to Δ-6D activity in the testicle and Elovl5 activity in the kidney.^134^ Higher levels of omega-3 PUFAs were produced compared to omega-6 PUFAs but levels may differ depending on the physiological or pathological condition of a patient. Both processes (desaturation and elongation of PUFAs) are influenced by the availability of zinc, vitamin B, and magnesium, protein levels in the diet, and oxidative stress in the liver.^135^ Obese individuals and those suffering from nonalcoholic fatty liver disease (NAFLD) produce less PUFAs.^136^

The intermediates formed as a result of Elovl5 activity were similar in omega-3 and omega-6 production. The activity of Elovl2 was higher with omega-3 substrates (EPA and stearidonic acid, SDA) compared with omega-6 substrates (ARA and adrenic acid, ADA), as observed with recombinant *Saccharomyces cerevisiae* cells that expressed Elovl2.^137,138^ A possible explanation for this is that the fatty acid (FA) transport protein 2a/very long chain acyl-CoA synthetase 1 (FATP2a/Acsvl1) enhances the transfer, activation, and metabolism of omega-3 PUFAs.^139^ This may lead to an increase in dietary DHA but depends on the availability of ALA, the elongation and/or desaturation of DHA precursors, and a range of other physiological and enzymatic conditions (summarized by Valenzuela *et al*.^134^

The synthesis of PUFA is initiated by the conversion of ALA and LA to an acyl-CoA derivative by acyl-CoA synthases 3 and 4, the desaturation of acyl-CoA by Δ6D and Δ5D to form a double bond, elongation (the addition of two carbon atoms) of PUFA acyl-CoA by elongase 2/5, and the oxidation of fatty acids (FAs) by peroxisomal FA oxidase (FAO).^135^ The end products EPA, DHA, and AA are important in cell growth, membrane formation, and the functioning of organs. The transcription of desaturases and elongases in mammals is regulated by insulin *via* the sterol regulatory element binding protein 1c (SREBP-1c), under control (suppression) by omega-3 PUFAs. For further information on the synthesis of omega-3 and omega-6 PUFAs, and the influence of nutritional status on the desaturation and elongation of these fatty acids, the reader is referred to Videla *et al*.^135^

## Saturated fatty acids (SFAs)

Saturated fatty acids (SFAs) are distinguished from unsaturated fatty acids by having single C – C bonds. Short-chain SFAs (C8 to C12) are found in vegetable oils, whilst SFAs with more than 12 carbons, e.g., palmitic acid and stearic acid (C_18_H_36_O_2_) are predominantly in eggs, animal fats, and butter^87^ SFAs are generally pro-inflammatory.^128^ The interaction of palmitic acid and other dietary SFAs with the nucleotide-binding oligomerization domain-leucine-rich repeat-pyrin domain-containing 3 (NLRP3) inflammasome leads to an increase in adiposity.^128^ Macrophages in adipose tissue have higher levels of the NLRP3 inflammasome, as observed in obese mice and humans. A decrease in NLRP3 inflammasome was noted when calorie intake was restricted or with an increase in exercise.^140^ *In vitro* studies have shown that diets rich in SFAs can activate TLR4 in dendritic cells and lead to an increase in NLRP3 inflammasome.^140^ Studies with human monocytes have shown that palmitate, myristate, and stearate, but not unsaturated fatty acids such as palmitoleate and oleate, activates TNFα and IL-1β, which promote death and increases inflammation.^128^ Palmitate stimulates the production of the inflammatory caspase proteins caspase-1, caspase-4, and caspase-5.^87^ These proteins play an important role in the production of IL-1β and the initiation of cell death.^141^ Palmitic acid, stearate, and lauric acid are known to regulate inflammatory responses via TLR4 and NFκB signaling in immune cells.^87^ The myeloid differentiation primary response 88 protein (Myd88) transduces signals from all TLRs, except TLR3.^87^ The toll/interleukin-1 receptor (TIR) domain contains the TIR adaptor-inducing beta interferon (TRIF) that sends signals from TLR3 and TLR4.^87^ TRIF protects cells from metabolic disorders and inflammation.^142^

Palmitic acid targets the receptor-interacting protein kinase 1 (RIPK1) in liver macrophages, leading to increased production of inflammatory cytokines (IL-1β, TNFα, and IL-6) and cell death. The condition is known as nonalcoholic steatohepatitis (NASH).^143^ Obese individuals and those suffering from type 2 diabetes are especially vulnerable to developing NASH. An increase in palmitic acid leads to autophagy and cellular accumulation of autophagosomes.^144^ Mice lacking the ability to produce the mixed lineage kinase domain-like protein (MLKL) were protected from autophagy when they were on a Westernized diet. They showed a reduction in liver injury, inflammation, and cell death.^138^ Palmitic acid induces the hypoxia-inducible factor-1α (hif-1 α), responsible for inflammation regulated via the NFκB pathway and the production of pro-inflammatory cytokines TNF, IL-1β, and IL-6.^145,146^

SFAs and a high-fat diet influence cellular processes in IECs, Paneth cells, and stem cells.^147,148^ Disruption of Paneth cells affects the production of antimicrobial peptides and growth factors that maintain stem cells. Previous studies have shown dysfunction in these cells in patients with IBD.^149^ In mice fed a high-fat diet, the dysfunction of Paneth cells led to the activation of type I interferons (IFNs) associated with nuclear farnesoid X receptor (FXR).^149^

Palmitic acid is converted to palmitoleic acid, oleic acid, stearic acid, and sphingolipids.^128^ Sphingolipids are also produced by bacteria, e.g., *Bacteroides fragilis*.^150^ Palmitic acid stimulates IgA responses, which may lead to the forming of mucosal adjuvants.^151^ Hepatocytes treated with palmitic acid release lipotoxic extracellular vesicles filled with sphingosine 1-phosphate (S1P). This stimulates the infiltration of macrophages and induces hepatic lipotoxicity associated with NASH.^152^

## Can fatty acids be used in the treatment of diseases?

Fewer cases of CVDs were reported for patients following a MD.^153^ A low-fat diet supplemented with PUFAs reduced waist circumference, blood pressure, triglyceride levels, and the prevalence of metabolic syndrome.^154^ The relative abundance of *Lachnospiraceae* associated with an MD was inversely correlated with blood pressure and lipid profiles.^155^ Oleic acid was associated with an increase in the *Clostridiales* vadin BB60 group.^155^ Tryptophan, an essential amino acid found in a variety of foods, including poultry, fish, dairy products, and grains,^156^ typical of an MD, is metabolized by gut microbiota into small molecules that serve as ligands for AhR. This stimulates the secretion of glucagon-like peptide 1 (GLP-1) from EECs.^156,157^ Intestinal barrier functions are impaired with reduced AhR and less GLP-1 being released.^157^ Tryptophan produced by gut microbiota promotes the differentiation of neural progenitor cells into mature neurons^158^ and reduces inflammation of the CNS.^159^ Although the consumption of seafood reduces the risk for CVD,^160^ the production of TMA by gut microbiota and the conversion to TMAO accelerates CVD, as shown in mice.^145,161^ A vegetarian diet, on the other hand, favors alpha bacteria,^162^ especially SCFA-producing taxa such as *Akkermansia*,^163^ *F. prausnitzii*, *Eubacterium rectale* and *Eubacterium biforme*. ^164^

Lauric acid, retinoids, tocotrienols, and carotenoids in palm oil enhance gut immune homeostasis by modulating the production of IgA.^116^ Retinoic acid (vitamin A) triggers the production of IgA in B cells.^165^ Food rich in biotin (vitamin B7), such as Yam (orange sweet potato) supports the proliferation and maintenance of gut microbiota that prevents the activation of NFκB and stimulates the generation of pro-inflammatory cytokines such as tumor necrosis factor α (TNFV), IL-8, IL-6, and IL-1.^166^ The antioxidative, immunomodulatory, and anti-inflammatory properties of vegetable flavonoids protect the host against chronic inflammatory diseases.^167,168^ Innate immunity and the constant production of neutrophils are important in sustaining a balanced gut microbiome^166^ and fight off invading microorganisms.^169^

Inulin-type fructans (ITFs), typically found in wheat, onion, and chicory,^170^ repress appetite^170^ and prevent constipation.^171,172^ Inulin stimulates the growth of *Bifidobacterium, Anaerostipes, Bacteroides*, and *Faecalibacterium* but represses the growth of *Coprococcus*, *Dorea*, *Ruminococcus, Bilophila, Blautia*, *Oscillibacter*, and *Ruminococcus*.^172–174^ Although inulin does not affect the production of SCFAs,^167,168^ changes were noted in the plasma levels of tyrosine and glycine.^174^ Inulin propionate ester (IPE) reduced the production of IL-8, increased the secretion of insulin,^174,175^ and stimulated the growth of *Bacteroides uniformis* and *Bacteroides xylanisolvens* but repressed the growth of *Eubacterium ruminantium* and *Blautia obeum*. ^174^

## Conclusions

Fatty acids are major constituents of cell membranes but are often overlooked as intracellular signaling molecules and gene expression modulators. In the past, most research on fatty acids focused on human health, especially CVDs, cancer, type 2 diabetes, and inflammatory diseases. Extensive research has been done on PUFAs, especially butyrate, and its role in IBD and CRC. Research on SCFA transports has shown that the dysregulation of monocarboxylate transporters such as MCT1, MCT4, and SMCT1 may be the answer to some gastrointestinal disorders. Acetate and propionate have similar notable effects on the GIT, with the latter demonstrating a pivotal role in weight management and the regulation of inflammation. The supplementation of a fiber-rich diet with SCFAs helps to maintain a healthy intestinal barrier and support diverse gut microbiota. More research is, however, required to explore the role intestinal microbiota play in the metabolism of SCFAs, including the mechanisms involved in the lowering of LDL-cholesterol by PUFAs such as omega-6, and the lowering of triglycerides by omega-3 PUFAs EPA and DHA. We need to understand the role of SCFAs in regulating blood flow, thrombosis, and neurological disorders. The relationship between omega-6 and omega-3 PUFAs in inflammation regulation is not fully understood. Even-numbered saturated fatty acids, such as palmitic acid, raise total and LDL cholesterol levels. Reports of saturated fatty acids that increase coagulation, inflammation, and insulin resistance necessitate in-depth research. The replacement of saturated fatty acids in a diet by *cis* MUFAs, such as palmitoleic and oleic acids, and ω-6 PUFA (LA) lower LDL cholesterol levels and are associated with fewer incidences of CVDs. Arachidonic acid, also a ω-6 PUFA, mainly acts as an eicosanoid precursor involved in inflammatory reactions but EPA and DHA are important mediators in signal transduction and gene expressions. *Trans* SCFAs raise LDL and lower HDL cholesterol levels, thus increasing the risk of CVD. *Trans* SCFAs also promote inflammation and are prone to play a role in metabolic diseases.
